# Recurrent Oral Cancer: Current and Emerging Therapeutic Approaches

**DOI:** 10.3389/fphar.2012.00149

**Published:** 2012-07-30

**Authors:** Sabrina Daniela da Silva, Michael Hier, Alex Mlynarek, Luiz Paulo Kowalski, Moulay A. Alaoui-Jamali

**Affiliations:** ^1^Department of Otolaryngology-Head and Neck Surgery, Sir Mortimer B. Davis-Jewish General Hospital, McGill UniversityMontreal, QC, Canada; ^2^Department of Medicine, Segal Cancer Centre and Lady Davis Institute for Medical Research, Sir Mortimer B. Davis-Jewish General Hospital, McGill UniversityMontreal, QC, Canada; ^3^Department of Oncology, Segal Cancer Centre and Lady Davis Institute for Medical Research, Sir Mortimer B. Davis-Jewish General Hospital, McGill UniversityMontreal, QC, Canada; ^4^Department of Head and Neck Surgery and Otorhinolaryngology, AC Camargo HospitalSão Paulo, São Paulo, Brazil

**Keywords:** oral cancer, recurrence, drug resistance, novel therapeutics

## Abstract

Oral cavity cancer (OCC) is associated with high incidence of loco-regional recurrences, which account for the majority of treatment failures post-surgery and radiotherapy. The time-course of relapse manifestation and metastasis are unpredictable. Relapsed OCC represents a major clinical challenge in part due to their aggressive and invasive behaviors. Chemotherapy remains the only option for advanced OCC whenever salvage surgery or re-irradiation is not feasible, but its efficacy is limited as a result of the drug resistance development. Alternatives to use of different permutations of standard cytotoxic drugs or combinations with modulators of drug resistance have led to incremental therapeutic benefits. The introduction of targeted agents and biologics against selective targets that drive cancer progression has opened-up optimism to achieve superior therapeutic activity and overcome drug resistance because, unlike the non-selective cytotoxic, the target can be monitored at molecular levels to identify patients who can benefit from the drug. This review discusses the multifactorial aspects of clinical drug resistance and emerging therapeutic approaches in recurrent OCC, emphasizing recent advances in targeted therapies, immunotherapy, and potential relevance of new concepts such as epithelial-mesenchymal transition and cancer stem cell hypothesis to drug resistance.

## Oral Cancer Recurrence and Therapeutic Modalities

Oral cavity cancer (OCC) is the most common site of malignancy in the head and neck being ranked as the eightieth most frequent cancer worldwide (Petersen, 2009) with over 145,500 deaths per year^1^ [International Agency for Research on Cancer (IARC; 2011)]. The main risk factors are exposure to exogenous carcinogens such as tobacco smoke and excessive alcohol consumption. The incidence varies among geographical regions, e.g., high incidence is reported in India, France, and South/Southeast Asia (Parkin et al., 2005; Su et al., 2006). OCC has a high occurrence of clinically occult ipsilateral or bilateral lymph node metastasis due to the rich lymphatic submucosal plexus that freely communicates across the midline facilitating the spread of neoplastic cells to any area of the neck (Fan et al., 2011). Most patients with OCC are diagnosed with tumors at advanced stage and incur significant morbidity and mortality due to the disease as well as sequels associated with therapeutic management and complications (Kowalski et al., 1998, 2005). The overall survival rate decreases as the carcinoma stage increases, from 75 to 90% for Stage I to 10–22% for Stage IV (Kowalski et al., 2005). The prognosis depends on tumor primary site, nodal involvement, tumor thickness, and the status of the surgical margins (Garzino-Demo et al., 2006). Conventional treatment for OCC includes surgery, radiotherapy, and chemotherapy. OCC surgical management often can lead to severe morbidity due to disfiguring and functional side effects (Furness et al., 2011). Surgery combined with chemotherapy and radiotherapy can improve overall survival, particularly in patients with advanced oral cancers. Induction chemotherapy may prolong survival by up to 20% and adjuvant concomitant chemoradiotherapy can improve survival by up to 16% (Furness et al., 2011). However, approximately one-third of patients treated with surgery and adjuvant therapy will experience local or regional recurrence and/or distant metastasis (Greenberg et al., 2003).

Local and regional recurrences account for up to 90% of treatment failures post-surgery and radiotherapy (Leemans et al., 1994; Carvalho et al., 2005; Agra et al., 2010). The rates of OCC recurrence vary from 18 to 76% for patients who underwent standard treatment, and it is considered the major cause of poor survival rates. Most studies corroborated that the median time to recurrence is 7.5 months after treatment, and 86% of the recurrences occur within 24 months (Carvalho et al., 2005; Kowalski et al., 2005; Fan et al., 2011). The presence of cervical lymph nodes metastasis is the most important adverse prognostic factor in OCC patients. Extracapsular spread is a particularly reliable predictive factor of loco-regional recurrence, distant metastasis, and death from disease (Greenberg et al., 2003). In this subset of patients, adjuvant chemoradiation proved to reduce the rates of recurrences when compared to radiation alone (Bernier et al., 2008). The histological status of surgical margins is another available assessment for recurrence risk in OCC (Leemans et al., 1994). Woolgar et al. (1999) reported that ∼10% patients with oral squamous cell carcinoma develop recurrence and the relapse appears much earlier than metachronous disease carrying the worst prognosis. Patients with recurrent carcinomas pose a clinical challenge with regard to defining the best therapeutic options. Only a small group of patients are candidates for salvage surgery and about 30–45% of these have poor survival rates (Agra et al., 2010). Patients who are not candidates for salvage surgery or re-irradiation usually receive chemotherapy, but even with the most recent combinations of drugs the prognosis remains poor and cure is rare (Vermorken et al., 2007). Clearly, new therapeutic options for recurrent OCC are urgently needed. A significant problem in those cases is postoperative and postradiotherapy fibrosis that precludes achieving adequate pharmacologic doses of the drug in the recurrent tumor site.

## Resistance to Chemotherapy Drugs in OCC

The most common chemotherapy drugs used for advanced OCC include taxanes (paclitaxel and docetaxel), anthracyclines (adriamycin, epirubicin, and pirarubicin), platinums (cisplatin and carboplatin), and antimetabolites (e.g., methotrexate, and 5-fluorouracil). Anthracyclines act primarily by interfering with DNA replication via interaction with topoisomerases, intercalation, and induction of DNA strand breaks. Platinum-containing compounds in particular cisplatin and carboplatin target DNA forming intra-strand and inter-strand cross-links causing distortion of the DNA helix and apoptotic cell death. Taxanes such as paclitaxel and taxotere, and vinca alkaloids such as vinorelbine and vincristine all interfere with microtubule and spindle assembly. Topoisomerase inhibitors (e.g., etoposide, topotecan, irinotecan) induce DNA strand breaks, while antimetabolites (e.g., gemcitabine, 5-FU, capecitabine, trimetrexate) are potent inhibitors of RNA synthesis. Exposure of cancer cells to chemotherapeutic agents culminate in the activation of tumor cell apoptosis and most are preferentially active on proliferating “cycling” cancer cells compared to “resting” normal cells making malignant cells marginally susceptible to these agents (Alaoui-Jamali et al., 2004). However, host toxicity can result in part through affect on normal bone marrow stem cells and epithelial cells that are mitotically active and hence susceptible to non-desirable cytotoxic effects of chemotherapeutic agents.

Empiric clinical trials have defined the standard first-line chemotherapeutic regimens and dosage that achieve the best therapeutic efficacy and with acceptable host toxicity for each tumor type. Examples of combinations include cisplatin-containing regimens for non-small cell lung cancer (NSCLC); 5-FU, leucovorin, and irinotecan for colon cancer; and anthracyclines, carboplatin, and paclitaxel for breast cancer. In advanced OCC, conventional cytotoxic drugs most commonly used include methotrexate, cisplatin, carboplatin, 5-FU, paclitaxel, and docetaxel (Specenier and Vermorken, 2010). Whether given alone or in combination, these chemotherapy drugs have produced clinical benefits in terms of substantial improvement of the overall survival in OCC patients when compared to other cancers notorious to be refractory to chemotherapy such as colorectal and renal carcinomas (Lebwohl and Canetta, 1998; Jassem, 1999; Giaccone, 2000). However, even in the most chemotherapy responsive cases, patients inevitably experience tumor progression or relapses due to the development of cells with acquired drug resistance, or emergence of cell subpopulations genetically refractory to the drugs (intrinsic drug resistance; Greenberg et al., 2003). Although there is a relatively common pattern of genomic abnormalities including DNA allelic loss during OCC progression from premalignant to malignant phenotype such as chromosomal losses at 3p, 9p, 17p, and mutations in *TP53*, supporting a distinct biology for OCC (Mao et al., 1996; Mydlarz et al., 2010), drug resistance is predictable given the common presence of genomic instability that can result in the accumulation of multiple genetic aberrations including those that impact chemotherapy response signaling (Califano et al., 1996, 2000; Weber et al., 1998; Okafuji et al., 2000).

The limitation of chemotherapy has been ascribed primarily to mechanisms that mediate drug resistance at the cellular level or factors innate to tumor microenvironment and the host. For instance, a variety of intracellular mechanisms have been associated with decreased drug transport or increased efflux including overexpression of plasma membrane efflux transport proteins (e.g., P-glycoprotein-170, Pgp170; MDR-related proteins – MRP; lung resistance protein – LRP) that prevent drugs from reaching intracellular targets (Trédan et al., 2007). Other mechanisms involved in drug metabolism and cellular response to DNA damage are equally important in the development of drug resistance; these include enhanced drug detoxification via upregulation of phase II detoxifying enzymes (e.g., glutathione *S*-transferases), enhanced DNA repair mechanisms that counteract drug-induced DNA damage, mutations in drug target-encoding genes that reduce the affinity of a drug to active site (e.g., mutations in tubulin and topoisomerase-coding genes that reduce taxanes and camptothecin activity, respectively), as well as a plethora of other mechanisms that make the cells more resistant to pro-apoptotic signals ranging from aberrant function of growth factor receptors and tumor suppressors to deregulated intracellular transduction pathways, cell cycle checkpoints, and chromatin and transcriptional modifications (Rudolf and Cervinka, 2003).

**Figure 1:**
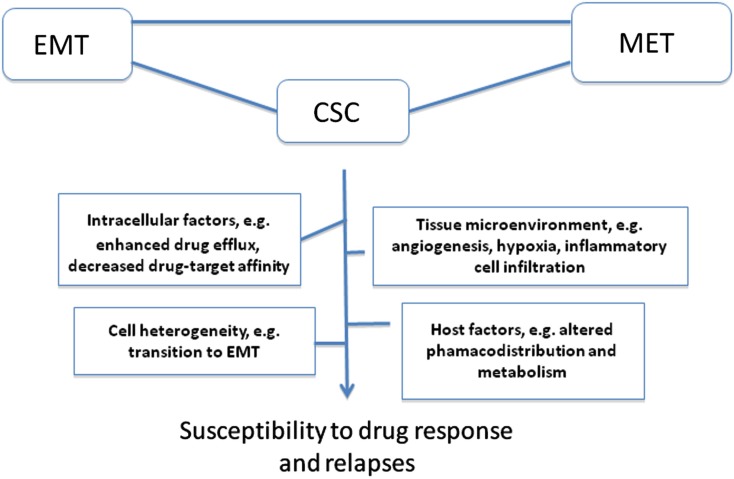
**Representative important factors and concepts implicated in drug resistance and relapses**. Various intracellular factors can account for impaired OCC cell response to chemotherapy drugs, including changes in the bioavailability of a drug or its active metabolites at the target site (decreased uptake or increased efflux), inability of cells to repair DNA damage which can lead to increase tolerance, and defects in cell ability to signal DNA damage response to downstream effectors targets to trigger cell death. Altered drug pharmacodistribution and pharmacodynamics in the host also impact on drug response. The selective pressure exerted by drugs combined with tumor cell heterogeneity (often a result of tumor genomic instability) is also a driving force for drug resistance. Tumors can develop resistance via regulation of their microenvironment, e.g., by remodeling the extracellular matrix, deregulating cancer cell-endothelial cell/immune infiltrating cell interactions, leading to enhanced angiogenesis, hypoxia, and resistance to cell death. In this context, epithelial-mesenchymal transition (EMT) and the reverse process mesenchymal-epithelial transition (MET), both are critical process for cancer progression to metastasis and homing in distant site contribute to drug resistance via various mechanisms, including induction of cell heterogeneity and selection of rare cancer stem cell (CSC) variants with intrinsic resistance to chemotherapy.

Tumor microenvironment is critical determinant and modifier of therapeutic response; in particular angiogenesis and hypoxia have been extensively investigated as alternatives to overcome drug resistance (Folkman, 2007; Sebens and Schäfer, 2011; Maione et al., 2012; Semenza, 2012). To reach tumor cell sanctuaries in primary or distant tissue targets, anticancer drugs must be delivered efficiently through the tumor vasculature, cross the vessel wall, and diffuse within tumor tissue. This would argue that pro-angiogenic drugs would favor superior chemotherapy drug pharmacodistribution. However, approaches to inhibit angiogenesis have been investigated as alternatives to overcome drug resistance (Maione et al., 2012). In this context, the concept of metronomic chemotherapy dosing based on the use of repeated low-dose chemotherapy combined with antiangiogenic drugs has provided exciting results in preclinical models to minimize side effects of conventional chemotherapy and to improve therapeutic response in drug-resistant tumors (Browder et al., 2000; Hanahan et al., 2000; Bocci et al., 2002; Moreno Garcia et al., 2012) although the efficacy of this approach remains debated. While initial optimism targeting angiogenesis was based on the notion that endothelial cells are genetically stable and hence less prone to develop drug resistance, it is now documented that tumor blood vessels are instable and differ from their normal counterparts with respect to morphological characteristics, blood flow, leakiness, as well as structural abnormalities in the basement membrane and in pericyte activity (Morikawa et al., 2002; Kawamoto et al., 2012). This would explain the common occurrence of tumor endothelial cell resistance to antiangiogenic drugs, including the anti-VEGF antibody bevacizumab (Ma and Waxman, 2008). As noted above, inhibition of angiogenesis has a drawback as it may prevent drug distribution within tumor tissue. Moreover, unexpected observations from preclinical studies revealed that antiangiogenic drugs can either improve or worsen prognosis (Rapisarda and Melillo, 2012). The most obvious effect is the induction of cell hypoxia, a hallmark of tumor aggressiveness and resistant to chemoradiotherapy. For instance, recent study by Cooke et al. (2012) revealed that antiangiogenic agents have a beneficial decrease in the growth of primary tumors, but also can promote distant metastases and hence worsen prognosis. This was attributed to activation of cell invasion signaling, including epithelial-mesenchymal transition (EMT) and hypoxia in tumors depleted from tumor vessel-associated cells such as pericytes within the tumor microenvironment (Cooke et al., 2012). Hypoxia is well documented to promote resistance to chemotherapy and radiation (Braybrooke et al., 2000; Semenza, 2010, 2012). Hyperbaric oxygen (HBO) has been proposed to reduce tumor hypoxia by increasing the amount of dissolved oxygen in the plasma, however HBO is associated with significant adverse effects including oxygen toxic seizures and severe radiation tissue injury (Bennett et al., 2008). Tumor cell heterogeneity is another important aspect for chemotherapy failure once that chemotherapy targeting more sensitive tumor cell subpopulations can select rare variants with intrinsic resistance; this idea has been used to explain the intrinsic resistance of cancer stem-cells (CSC; Figure 1).

## EMT and CSC Concepts and Their Impact on Drug Resistance

Traditional cancer treatments were developed by virtue of their anticancer activity against a tumor mass cell population (e.g., log kill-based principle). The tumor mass represents several subpopulations of cells with distinct genotypes and phenotypes, including response to chemotherapy. It is recognized that tumor heterogeneity contributes by several mechanisms, including activation of the EMT. In this case well-differentiated cancer cells require a higher plasticity and invasive capacity via a conversion to non-polarized and poorly differentiated mesenchymal cells (Thiery, 2002). Activated EMT signaling pathways (e.g., activation of Wnt/β-catenin, PI3-K/AKT, MAPK, and Notch) is well documented to occur in advanced OCC (Boyer et al., 2000; Conacci-Sorrell et al., 2002; Nelson and Nusse, 2004; Larue and Bellacosa, 2005; Nawshad et al., 2005; Tommasi et al., 2007; Barker, 2008; Agarwal et al., 2010; Courtney et al., 2010; Falasca, 2010; Ihle and Powis, 2010; Wong et al., 2010). Preclinical studies also reported a significant correlation between EMT phenotype, drug resistance, and relapses (Zeisberg and Neilson, 2009) including in OCC patients (Machiels and Schmitz, 2011; Raza et al., 2011).

Equally important, the concept of CSC provided alternative frameworks to explain drug resistance and relapses. CSC is defined as a small subpopulation of tumor cells having both tumor-initiating ability and the ability to reconstitute the cellular heterogeneity of the original tumor. Several studies have implicated CSC in cancer progression, invasion process, loco-regional recurrence after therapy, and distant metastasis (Prince et al., 2007). Also, cancer cell variants expressing CSC markers have been reported to be more resistant to chemotherapy than cells that do not express CSC markers (Dean et al., 2005). This implies that chemotherapy drugs, by targeting the most sensitive non-CSC can contribute to enrichment of the chemotherapy-resistant CSC and hence promote recurrences (Frame and Maitland, 2011). This resistance may reflect the preservation of normal stem cell protective mechanisms, such as an increased expression of drug efflux pumps or alterations in apoptotic, cell cycle, and DNA repair mechanisms (Kvinlaug and Huntly, 2007). Interestingly, EMT which also promotes drug resistance is found to contribute to selection/enrichment of cell subpopulations with stem cell characteristics from well-differentiated epithelial cells (Mani et al., 2008; Polyak and Weinberg, 2009). Therefore several emerging approaches targeting key signaling molecules critical to CSC, and cancer epigenetics and metabolism are under extensive investigation (Grskovic et al., 2011; Vander Heiden, 2011; Arrowsmith et al., 2012).

## Emerging Therapeutic Options for Advanced OCC

Conventional chemotherapy is widely used for refractory OCC despite only incremental improvement in disease-free survival. The broad diversity of drug resistance mechanisms led to alternative approaches incorporating drug resistance modulators in the chemotherapy regimens. Possible mechanisms of acquired resistance include altered cellular drug transport (e.g., inhibitors of *P*-glycoprotein 170 encoded by the *MDR1*), enhanced intracellular detoxification, increased DNA repair, and enhanced tolerance to DNA damage (Persons et al., 1999; Garraway and Jänne, 2012). However, this approach has limited success given the large number of genetic changes affecting multiple cell regulatory pathways that regulate drug response. Moreover, recurrent OCC treated by chemotherapy often expresses an aggressive progression and develop cross-resistance to a wide range of structurally and mechanistically unrelated drugs (Garraway and Jänne, 2012). This is a limitation in the use of alternative therapeutic combinations since the process of selecting treatment has been based on the availability of chemotherapy agents, as well as different drug modes of action.

The great progress in the understanding of cancer biology has led to the development and validation of several target-selective agents referred as targeted therapies with improved efficacy against chemotherapy refractory cancers. These classes of drugs have selectivity toward targets proven to play a rate-limiting step in the process of cancer progression and many are in the clinical use for specific cancers, e.g., EGFR-tyrosine kinase inhibitor Tarceva (Erlotinib for invasive NSCLC and pancreatic cancer (Rosell et al., 2012; Troiani et al., 2012); anti-EGFR monoclonal antibody Cetuximab (Erbitux) for colorectal carcinoma (Debucquoy et al., 2010); the monoclonal antibody Trastuzumab (Herceptin) for HER-2/neu+ breast cancer (Chang, 2010); several B-Raf inhibitors for melanoma (Lott, 2011); antiangiogenesis agents such as the anti-VEGF monoclonal antibody Bevacizumab (Avastin) for metastatic cancers of the lung, colon, and kidney (Kerr, 2004); the proteasome inhibitor Bortezomib (Velcade) for multiple myeloma (Mahindra et al., 2012); the histone deacetylase inhibitor Vorinostat (Zolinza) for cutaneous lymphoma (Lansigan and Foss, 2010); and the Bcr-Abl inhibitors including Gleevec for chronic myeloid leukemia (CML). In the case of OCC, the clinical use of targeted agents is still lagging behind but ongoing multi-institutional clinical trials are being conducted to investigate their utility compared to conventional cytotoxic chemotherapy (Table 1). Given that most of the targeted agents cited above, including those targeting EGFR and HER-2 receptors, VEGF and VEGFR, Raf, and proteasome, are also deregulated in OCC, they may provide potential therapeutic benefits for patients with advanced OCC. Moreover, a current alternative in oncology research is to improve the therapeutic efficacy of existing chemotherapy agents focusing on combination of targeted agents with cytotoxics drugs, or their use as long-term maintenance therapy (either low dose/high frequency of standard cytotoxic drugs or molecularly targeted agents like Avastin, Herceptin, Erlotinib) following high-dose induction therapy, the initial treatment used to reduce tumor size^2^ [National Cancer Institute at National Institutes of Health (NCI; 2012)]. In this way, OCC could certainly benefit from advanced funding from other cancer types particularly because the suitability of OCC for multiple biopsies that can aid target profiling and patients selection.

**Table 1:** **Recent drugs, targets, and clinical trials in head and neck cancers**.

Class of drugs	Commercial name	Target	Head and neck clinical trials
E7080		VEGFR2	Phase II
Erlotinib	Tarceva	EGFR	Phase III
Gefitinib	Iressa	EGFR	Phase II
Imatinib	Gleevac	PDGFR, BCR-ABL, KIT	Phase II
Lapatinib	Tykerb	EGFR	Phase III
Pazopanib	Votrient	VEGFR, PDGFR, KIT	Phase II
Sorafenib	Nexavar	VEGFR, PDGFR, KIT, FLT-3 RAF	Phase II
Sunitinib	Sutin	VEGFR, PDGFR, KIT, FLT-3 RET	Phase II
Vandetanib	Zactima	VEGFR, RET,EGFR	Phase II/III
XL-184		MET, VEGFR, RET	Phase III
Bexarotene	Targretin	RXR	Pilot study
Irofulven + capecitabine	MGI-114 + Xeloda	p53, MDR1	Phase III
Bevacizumab	Avastin	VEGF	Phase III
Cetuximab	Erbitux	EGFR	Phase III
Nimotuzumab	BIOMAb EGFR	EGFR	Phase III
Panitumumab	Vetibix	EGFR	Phase II
Trastuzumab	Herceptin	ErbB2	Phase II
Zalutumumab	HuMax-EGFr	EGFR	Phase III
Temsirolimus	Torisel	mTOR (PI3k)	Phase II
Bortezomib	Velcade	26s Proteasome inhibitor	Phase II
Valproic		Acid epigenetic alterations	Phase II
Oxaliplatin	Eloxatin	Induction of Bax/Bak	Phase II
Docetaxel	Taxotere	Microtubules	Phase III

*(NCI www.cancer.gov/clinicaltrials website)*.

## Conclusion and Perspectives

The efficacy of conventional cytotoxic chemotherapy for OCC has been hampered by lack of selectivity, narrow therapeutic margin, and the common development of drug resistance mechanisms. Due to the broad multifactorial aspect of drug resistance phenotype, it is not surprising that the initial optimism surrounding modulation of a single drug resistance marker to overcome resistance in several other cancers has waned. Likewise, advances in molecular biology and drug discovery technologies have led to the development of pharmacologic agents and therapeutic antibodies that selectively target crucial signaling molecules with preferential expression on malignant cells, or their surrounding tissue microenvironment. Clinical experience with several targeted drugs, including small molecules and therapeutic antibodies, revealed agents that can be better tolerated and it offers advantage in the identification of subsets of patients who can be benefit during the treatment. Furthermore, the shift toward targeted agents has potential to establish a novel framework, which can be used to develop alternative strategies to identify druggable tumor target. These targets can be adapted to individual cases with greater potential to counter critical drug resistance mechanisms (Garraway and Jänne, 2012), e.g., specific mutations in EGFR in NSCLC or B-Raf in melanoma can predict either sensitivity or resistance. Equally important and as described above, unexpected observations from preclinical studies clearly support the necessity to evaluate carefully the endpoints used to analyze the therapeutic utility of targeted agents. Targeted agents such as antiangiogenic drugs may lead to double edged effects by either improving or worsening prognosis (Cooke et al., 2012; Rapisarda and Melillo, 2012).

It is clear that a better understanding of the molecular and biological profile of OCC should facilitate the development of more efficient targeted therapies. Current clinical trials with targeted agents in OCC are likely to bring promising directions decreasing the risk of tumor recurrence and improving survival of patients with advanced OCC. Equally important, OCC represent an interesting clinical model to address translational aspects of drug relapses and new agents. Unlike cell lines and transplantation models, which revealed limited predictive clinical value possibly due to genetic and physiological differences between animal and human (Gillet et al., 2011), OCC is generally an accessible disease since multiple biopsies are feasible compared to other tumors location. This would allow for monitoring predictive molecular targets of utmost importance to clinical drug response versus relapses. These are critical issues for the development of efficient personalized therapy and in identification of novel therapeutic targets as well as monitoring the course and status of the disease.

## Conflict of Interest Statement

The authors declare that the research was conducted in the absence of any commercial or financial relationships that could be construed as a potential conflict of interest.

## Footnotes

^1^http://www.iarc.fr/study_booklptrivandrum.php

^2^http://www.cancer.gov/dictionary?cdrid=45736NCI
